# Malignancy in Dialysis Patients—How Serious Is the Problem, Especially in Relation to Waiting List Status?

**DOI:** 10.3390/cancers17172782

**Published:** 2025-08-26

**Authors:** Letycja Róg, Jacek Zawierucha, Bartosz Symonides, Wojciech Marcinkowski, Sławomir Jerzy Małyszko, Jolanta Małyszko

**Affiliations:** 1 Department of Oncology, Medical University of Warsaw, ul. Banacha 1A, 02-091 Warsaw, Poland; letycja.rog@uckwum.pl; 2 Fresenius Medical Care Polska S.A., 60-118 Poznan, Poland; jacek.zawierucha@fmc-ag.com (J.Z.); wojciech.marcinkowski@fmc-ag.com (W.M.); 3 Department of Internal Medicine, Hypertension and Vascular Diseases, Medical University of Warsaw, 02-091 Warsaw, Poland; bartosz.symonides@wum.edu.pl; 4 Department of Physiology and Pathophysiology, Medical University of Warsaw, 02-091 Warsaw, Poland; slawomir.malyszko@uskwb.pl; 5 Department of Nephrology, Dialysis, and Internal Medicine, Medical University of Warsaw, ul. Banacha 1A, 02-091 Warsaw, Poland

**Keywords:** malignancy, end-stage kidney disease, waiting list

## Abstract

**Background:** The overall incidence of malignancy in patients with end-stage kidney disease (ESKD) is reportedly higher compared to the general population. Cancer remains one of the dominant causes of death in these patients, due in part to uremia-induced impairment of tumor immune surveillance. Malignancy is one of the major limitations in the evaluation of potential kidney transplantation. This study aimed to assess the prevalence of cancer in hemodialysis population, particularly in relation to the waiting list. **Materials and Methods:** From the population of 5879 prevalent hemodialysis patients (60% men), 757 of them had a history of malignancy. In this population, 449 patients were actively waitlisted, and 4619 were not considered for potential kidney transplantation. Only 54 patients had unclear status in relation to active waiting list (during evaluation/disqualification). We assessed demographic data, basal biochemical data, and comorbidities, including malignancy, in relation to age, sex, presence of metastasis, and being actively waitlisted. **Results:** Malignancy was reported in 13% of hemodialysis patients, 6% of which had metastatic disease. Patients with malignancy were older (*p* < 0.001). More cases of cancer were observed in males (*p* = 0.02), who also had higher Charlson Comorbidity Index scores. Moreover, in patients with cancer, cardiovascular diseases were more common. They were also more malnourished (lower albumin, hemoglobin, lean mass) and more inflamed (higher ferritin, lower phosphorus). Only 27 patients with cancer were actively waitlisted, representing only 3.8% of this population. Patients with prior cancer on the active waiting list constituted 6% of all the waitlisted patients. Patients with a history of malignancy on the active waiting list were significantly younger, healthier, with significantly lower Charlson Comorbidity Index score, significantly lower ferritin, lower prevalence of diabetes, and higher blood pressure when compared to patients with malignancy who not listed for kidney transplantation. **Conclusions:** As malignancy became a more common comorbidity in dialysis patients, the elderly in particular, standardized cancer screening protocols should be promoted in dialysis units. Modern oncology has made huge progress, enabling the treatment of previously incurable cancers, as malignancy after kidney transplantation is considerably increased either due to de novo cancers or the recurrence of previous malignancy. Therefore, the evaluation of potential kidney transplant recipients, with tailored cancer screening and multidisciplinary evaluation, is strongly recommended. Besides a history of malignancy, the cardiovascular status also determines the eligibility for transplantation in dialysis patients. It is of paramount importance as the main cause of death in transplant recipients is cardiovascular death followed by malignancy.

## Simple Summary

Malignancy is a significant concern for patients on dialysis, and their status on the kidney transplant waiting list can influence the prevalence and management of cancers. Malignancy is less common in patients actively waitlisted than in to kidney transplant recipients. However, both groups have an increased risk of cancer compared to the general population. Waiting times on dialysis can also impact cancer risk, with longer waiting periods potentially increasing the risk of certain cancers and cancer-related deaths. When evaluating a patient for transplant, transplant centers must consider not only the risk of cancer recurrence or de novo malignancy, but also other potential complications associated with long-term dialysis, like cardiovascular disease.

## 1. Introduction

Chronic kidney disease (CKD) patients undergoing dialysis face a significantly higher risk of developing malignancies compared to the general population [1,2,3]. Kidney transplant recipients (KTRs) have a two- to three-fold increased cancer risk when compared with the general population [1,2,3,4]. More importantly, incident dialysis patients with a prior cancer history have a greater risk for cancer recurrence or developing a new cancer [5].

Cancer is the second cause of death in the dialysis population. Among dialysis patients, certain types of cancer, such as kidney, bladder, and thyroid malignancies, are particularly prevalent [2]. This increased susceptibility is attributed to a combination of factors, including chronic inflammation, impaired immune surveillance, and prolonged exposure to uremic toxins [6]. Therefore, before the transplantation, potential candidates must undergo special screening programs. The evaluation of potential kidney transplant recipient includes a search for possible contraindication for engraftment such as cardiovascular disorders, active infections/inflammation, and active and/or preexisting cancer [4].

However, the atypical presentation of cancer symptoms in this population often leads to a delayed diagnosis and poorer prognosis [7]. Moreover, the performance of screening tests in dialysis patients may differ from that in the general population, potentially affecting their sensitivity and specificity in cancer detection [8]. Due to the uncertainties surrounding cancer screening in patients undergoing long-term dialysis, a personalized approach is recommended. This approach should carefully weigh the patient’s life expectancy and the possible benefits of screening against its associated costs and potential risks [8].

Cancer and chronic kidney disease constitute two major public health burdens, and both are on the rise. Nevertheless, the number of patients affected simultaneously by both conditions is growing [9]. The overlap between oncology and nephrology is of growing importance [10], because these multidisciplinary teams face numerous challenges. Issues such as drug removal from hemodialysis and altered pharmacokinetics demand careful optimization of anticancer drug therapy, including dose adjustment and timing of administration. In addition, when peritoneal hemodialysis patients require anticancer drug therapy, they are often transferred to hemodialysis [11,12,13]. Another challenge is that multiple comorbidities, including diabetes and hypertension, along with the management of advanced CKD complications such as electrolyte imbalances, acid-base disorders, and fluid retention, can be significantly affected by various anticancer treatments [11].

Understanding the unique interplay between dialysis and cancer development is crucial for improving early detection, treatment outcomes, and overall patient survival. Further development of onconephrology may contribute to the preparation of schemes of management and administration of oncological drugs, radiotherapy, and surgery in dialysis patients. Transplantation, the optimal method for kidney replacement therapy, not only improves the quality of life but also prolongs survival compared with hemodialysis and peritoneal dialysis [14,15]. However, it is important to pay attention to the fact that performing kidney transplant in a patient with cancer has harmful effects of immunosuppression that can increase mortality and morbidity [16,17]. Patients with a pre-transplant malignancy require cancer-free waiting time between 2 and 5 years. The length of waiting time depends on the type of cancer and the risk of its recurrence [18,19,20].

It is important that kidney transplant candidates are carefully evaluated to detect and treat coexisting illnesses, including cardiovascular disease and malignancy, which may affect perioperative risk and survival after transplantation, as well as transplant candidacy. Thus, the evaluation should be as efficient and cost effective as possible. However, the rationale for cancer screening a potential kidney transplant recipient is to maximize the likelihood of a successful outcome following transplantation rather than to reduce the risk of death from cancer per se. Being actively waitlisted for patients with end-stage kidney disease is a chance for a longer life of better quality, but it also bears the risk of potential selection bias when compared to the dialysis population. In our two small previous studies conducted at a public university, we reported that only 6% of actively waitlisted patients had a prior history of cancer (3 out of 50) [21], whereas prior or current malignancy was observed in 22% of HD patients (24 out of 108) and 21% (10 out of 48) of peritoneally dialysis patients [22].

This study aimed to assess the prevalence of cancer in a large cohort of prevalent hemodialysis patients, particularly in relation to their waiting list status. In addition, we also presented the characteristics of the dialysis patients with malignancy.

## 2. Materials and Methods

From the population of 5879 prevalent hemodialysis patients (60% men, all Caucasians), from the biggest provider of hemodialysis in non-tertiary centers in Poland, i.e., Fresenius Medical Care, Poland, 757 of them had a history of malignancy. Among these 5879 patients, 449 were actively waitlisted, while 4619 were not considered for potential kidney transplantation. A total of 54 patients were excluded from further analysis due to unclear status on the waiting list (during evaluation/temporary disqualification, i.e., due to catheter-related sepsis, treatment of ongoing acute illness, etc.). We analyzed the database updated on 6 October 2022, including averaged data from a full 4-week period from 29 August 2022 to 25 September 2022 (it relates to blood pressure values, effective weekly treatment time, ultrafiltration, etc.). The characteristics of the patients are depicted in Table 1.

We assessed demographic data, basal biochemical data, cardiovascular disease prevalence, and dialysis vintage. We also added data on vascular access (arterio-venous fistula vs. tunneled cuff catheter). Apparent treatment-resistant hypertension was defined as blood pressure values ≥ 140/90 during treatment with 3 or more hypotensive drugs or the use of 4 hypotensive drugs regardless of blood pressure values. Cardiovascular disease (CVD) was diagnosed when at least one of the following conditions was present: heart failure, coronary artery disease (CAD), peripheral artery disease, and cerebrovascular disease. Data on presence or absence of malignancy and other comorbidities, as well as drugs, were retrieved from available files and reported in the computer system used in dialysis units.

### Statistical Analysis

Group differences in continuous variables were calculated using the *t*-test. Categorical variables were compared using the chi-square test. A two-tailed *p*-value less than 0.05 was considered statistically significant. The statistical analysis of the data was performed using R (R v4.1.2, R-core Team, R Foundation for Statistical Computing, Vienna, Austria, 2021, https://www.r-project.org).

The local ethics committee did not require ethical approval because the database was collected for a healthcare provider, and all data were fully anonymized before the authors had access to them. In addition, there was no direct patient contact.

## 3. Results

Among the dialysis population, malignancy as a comorbidity was present in 13% of the hemodialysis population studied. Metastatic cancer was present in 45 out of 757 (6%). Table 1 presents data collected from a group of 5879 patients who were dialyzed in different centers. A total of 757 of them had a history of malignancy. As we can clearly, see older age correlates with cancer occurrence. Patients with malignancy were older (*p* < 0.001). Cancer was more prevalent in males (*p* = 0.02); moreover, they had higher Charlson Comorbidity Index. Furthermore, patients with cancer more frequently suffer from cardiovascular diseases (however, both systolic and diastolic blood pressure was lower in cancer patients). There were no significant differences in BP medications (*p* > 0.05 for all classes), and no significant differences were observed in dialysis duration or weekly treatment effectiveness. Moreover, cancer patients on dialysis exhibit markers of malnutrition (lower albumin, hemoglobin, lean mass) and laboratory anomalies, which point to inflammation (higher ferritin, lower phosphorus). This can be a sign of cancer-associated cachexia or inflammation.

Older patients (>65 years) have higher Charlson Comorbidity score 633 (1.98), a greater prevalence of cardiovascular diseases 378 (70.7%), and diabetes 198 (37.0%), and lower systolic/diastolic blood pressure and heart rates (Table 2). Younger patients (<65 years) spend more time on dialysis and have higher ultrafiltration values. There are no significant differences in gender distribution, BMI, or metastatic solid tumors. It is important to note that older patients require tailored cardiovascular and diabetes management. Nevertheless, diuretics are used more often in recent malignancy (56.1% vs. 47.3%, *p* = 0.034), possibly addressing fluid overload. Worse nutritional markers such as lower phosphorus (4.39 vs. 4.89, *p* < 0.001) and lean tissue mass (33.1 vs. 37.0, *p* < 0.001) suggest muscle wasting. In addition, higher fat tissue indices (FTI: 14.7 vs. 13.3, *p* = 0.011) but similar BMI values suggest a sarcopenic obesity pattern.

Table 3 shows different groups of patients (N = 757), divided into women (F = 274) and men (M = 483). The average age differed significantly between the sexes (*p* = 0.049), whereas body mass index (BMI) showed no statistically significant difference (*p* = 0.052). It is important to note that a significantly higher percentage of men suffer from cardiovascular diseases (67.9% vs. 57.7%, *p* = 0.006). There is no significant difference in the frequency of diabetes in the individual groups. Male patients exhibit greater UF volumes, and lower adiposity, but similar dialysis outcomes. Female patients have lower Hb, DBP, and lean mass, along with higher FTI and MCV, partially explained by physiological norms; however, females may require closer monitoring for anemia (low Hb) and phosphorus levels, while males need CVD risk management.

The below Table 4 shows groups of patients divided according to the presence of metastatic malignancy. We can conclude three significant differences from the data. First, patients with metastases have a significantly higher rate of comorbidities, which suggests a greater health burden in this group of patients. In addition, patients with metastases have a shorter dialysis period and are less likely to use beta blockers. The shorter duration of dialysis in the group with metastases may suggest that these patients have a shorter life expectancy or a different course of treatment. In addition, the less frequent use of beta blockers in patients with metastases may indicate different treatment regimens in advanced cancer. In addition, the group with metastases has symptoms of cachexia (lower BMI, muscle and fat mass).

In Table 5, we present a characteristic of patients with cancer in relation to the waiting list status for kidney transplantation. Of note is that only 27 patients were actively waitlisted. All the actively waitlisted patients were cleared for transplantation by medical oncologist. From all the population studied, actively waitlisted patients with malignancy represented only 3.8%. (27 out of 708). Patients with prior cancer on the active waiting list constituted 6% of all the waitlisted patients (27 out of 449). Patients with a history of malignancy on the active waiting list were significantly younger, healthier, with a significantly lower Charlson Comorbidly Index score, significantly lower ferritin, lower prevalence of diabetes, and higher blood pressure when compared to patients with malignancy not listed for kidney transplantation.

## 4. Discussion

In our study, the prevalence of cancer was 13% in the hemodialysis population, whereas the prevalence of cardiovascular disease was 60%. Malignancy is more common among patients on hemodialysis than among those on peritoneal dialysis [23,24] and approximately three times more common in patients over 65 years old compared to younger patients, and less common in patients with diabetes, probably due to increased mortality from cardiovascular disease and other causes. In our population, malignancy was significantly more common in elderly patients (71% vs. 54%, *p* < 0.001); however, the presence of metastatic disease was similar. We could not find statistical difference in cancer prevalence in relation to dialysis vintage. However, HD patients with cancer were more inflamed and cachectic. However, data on the cancer prevalence in HD population is rather limited, particularly in relation to the waiting list. In Catalan cohort of 21,595 people on dialysis, the prevalence of cancer was 23.5%, much higher than in our population [1]. They also acknowledged including the absence of certain risk factors such as smoking, socioeconomic status, or treatments prior to dialysis.

In a recent study from Romania, conducted on a cohort of 1377 HD patients, cancer was diagnosed in 3.6% before the beginning of dialysis treatment and in 6.9% after the initiation of HD therapy [25]. They did not study the prevalence of malignancy in waitlisted patients. In the Multicenter Japan Cancer and DialYsis (J-CANDY) study, conducted in 20 hospitals during the period of 2010–2012, they identified 502 HD patients with cancer (the number of HD patients at that time was not provided) [26]. Chinnadurai et al. [27] reported that in the cohort of 1271 patients on dialyses between January 2012 and December 2017, at baseline 10.1% had cancer (previous or past) as comorbidity, with a 1.3% annual increase. These patients were significantly older, with higher prevalence of males, similar to our findings. Older data are coming from Europe [28], where among 7578 patients who initiated kidney replacement therapy in 2014 in 11 countries/regions in Europe, the prevalence of malignancy ranged from 5.6% in Spain (Aragon) to 20.2% in Dutch-speaking Belgium. The prevalence of malignancy as a comorbidity at the onset of renal replacement therapy increased between 2005 and 2014 (APC 2.0; 95% CI 1.0–2.5) mainly due to the rise in patients older than 65 years of age beginning renal replacement therapy relative to the general population.

Kidneys play a significant role in maintaining immune system homeostasis. Kidneys are responsible for clearing cytokines and bacterial antigens from the blood [29]. Chronic inflammation is marked by persistent tissue damage, cell proliferation in response to injury, and ongoing tissue repair promoting cancer development and progression [30,31,32].

In patients undergoing dialysis, the accumulation of uremic toxins due to impaired kidney function plays a significant role in increasing the risk of cancer development. These toxins exert systemic effects that can contribute to carcinogenesis through various mechanisms, including chronic inflammation [33] and cellular signaling pathways responsible for apoptosis (programmed cell death) and cell cycle regulation [34]. Immune dysfunction is another critical factor linked to uremic toxins. They can also promote angiogenesis—the formation of new blood vessels which supports tumor growth and metastasis [33,34].

Dialysis patients often experience chronic inflammation and oxidative stress, as mentioned before, which, when combined with immunosuppressive therapy, further increases the risk of DNA damage and cellular mutations, potentially leading to cancer. Statistically, the malignancy occurrence risk is increased with the increase in the total dose and time of immunosuppressive drugs use [35]. However, in our study, we could not assess possible relation between prior immunosuppression (as therapy for either glomerulonephritis, vasculitis, etc.) as data on this type of previous treatment were not available. We did acknowledge this as a gap and limitation.

Dialysis, an essential part of the treatment of end-stage renal failure, is associated with numerous health challenge. Patients with a history of cancer are less likely to be listed for kidney transplantation due to the increased risk of complications and disease recurrence. Cancer is one of the main contraindications to transplantation, because the immunosuppression necessary after the procedure can promote the development of cancer. Introducing organ transplantation in such patients requires special attention and careful monitoring to ensure optimal treatment outcomes. When summarizing the results of data analysis, it can be observed that waitlisted patients are a much healthier population with fewer comorbidities. However, despite high ultrafiltration during dialysis, blood pressure is over the target range for the present guidelines. The low number of hypotensive medications should be reassessed, and the treatment of hypertension requires further workup for better long-term outcomes.

In our previous study conducted on 300 potential kidney transplant recipients (40% females) from 26 dialysis units, representing 9.7% of all dialysis patients in facilities, we presented comorbidities in waitlisted vs. non-listed patients [36]. We did not include data on previous malignancy in the waitlisted population. However, recently diagnosed malignancy or additional diagnostic procedures were a cause of being non-listed in 10% of the entire non-listed population. In our study, only 7.6% of the patients were actively waitlisted.

KDIGO guidelines [18] recommend routine cancer screening, as per local guidelines for the general population as a part of the evaluation of potential kidney transplant recipients. However, the quality of evidence is very low (D). According to these KDIGO guidelines, it is recommended that patients with active malignancy be excluded from kidney transplantation, except for those with indolent and low-grade cancers, i.e., prostate cancer (Gleason score ≤ 6), superficial non-melanoma skin cancer, and incidentally detected renal tumors (≤1 cm in maximum diameter) (1B). As the mean age of potential transplant recipients increases, the rate of transplant recipients with history of malignancies also rises. In our previous review we discussed cancer screening in potential kidney transplant recipients based not only on KDIGO but also on the more recent consensus expert opinion statement from the American Society of Transplantation [19]. In the study published in 2005, Fischereder and Jauch [37] determined the prevalence of malignancy in patients considered for renal transplantation. The prevalence of cancer in potential kidney transplant recipients was 9.9%, much higher than in our patients on the waiting list. In their study, the mean time from cancer diagnosis was 2.2 years for patients who were actively waitlisted. In Poland the active waiting time is less than one year. It may explain the differences in cancer prevalence on the active waiting list. Longer waiting times may pose a risk of increased malignancy development and diagnosis, as well as reduced chances of therapy with curative intent. On the other hand, remaining on dialysis is associated with a 5% yearly mortality rate [38]. In a large European cohort of 9722 incident HD patients (data from Fresenius Medical Care), any history of cancer has been shown to increase mortality (HR = 1.75, 95% CI = 1.49–2.05 at 2 years) [39]. Therefore, waiting list status is a critical variable as a risk factor for mortality [40]. However, according to the 23rd Eurotransplant annual report, first year mortality on the active waiting list is 3% [41], whereas for patients on dialysis, it reaches 14%. Mortality was higher for patients over 65 years of age [42], but cancer was not included as a comorbidity. On the other hand, among solid-organ transplant recipients in the US, in the period of 1987–2018, 13% of deaths were caused by cancer [43].

Surprisingly, data on the prior malignancy on the active waiting list is scarce. Similarly, limited data are available on the epidemiology of cancer in both prevalent and incident patients on kidney replacement therapy. In our previous study [22] from our dialysis unit, among seven waitlisted patients (out of one hundred and fifteen on dialyses), only one had a history of malignancy (14% of all waitlisted patients). In this study we focused on the malignancy prevalence in the largest available database in Poland and the prevalence of malignancy in waitlisted patients.

A strength of our study is the high volume of an unselected patient population, representing one third of all hemodialysis patients in Poland, all treated under the same standardized protocol and using the same equipment by one of the largest European dialysis providers. The population of 5879 hemodialysis patients analyzed in relation to waiting list status can be considered the largest database of patients in Poland examined to date. Consequently, it is not only it can be representative of Poland but also of the broader Central and Eastern European region. In addition, it reflects real-world data, with all its inherent benefits and limitations.

## 5. Limitations

The limitations include the lack of a cancer detailed diagnosis, staging, and therapy and the lack of data on previous immunosuppressive therapy or smoking history. However, the presence of COPD is provided (in the Euclid system, only the information about cancer history and the presence of metastatic disease is provided). We are fully aware that a major gap is the lack of data on the type of malignancy. Only ICD-10 were included in the system to mark the diagnosis of malignancy. The rest of the data were input by attending physicians; therefore, detailed information is missing. We are fully aware that having more detailed data would allow for better assessment of clinical characteristics of the dialysis population with a history of malignancy.

## 6. Conclusions

To summarize, patients on the waiting list still represent a carefully selected and healthier dialysis population. This undoubtedly introduces a selection bias. Despite limited data on the optimal management of ESRD patients with cancer, it is essential that patients with chronic kidney disease and a previous or current history of malignancy should be assessed by a multidisciplinary team, taking into account cancer history, comorbidities, waiting time, and the chosen modality of kidney replacement therapy. The presence of malignancy should not be a limitation for access to renal replacement therapy, including transplantation. As malignancy becomes an increasingly common comorbidity in dialysis patients, particularly in the elderly, standardized cancer screening protocols should be implemented in dialysis units. In addition, in an aging population, the decision to evaluate a potential transplant candidate with a history of malignancy should be made collaboratively by the medical oncologist and the transplant physician. It is important to remember that cardiovascular disease, a common comorbidity in dialysis patients, also contributes to mortality risk, and these risks must be considered alongside cancer risks when evaluating transplant eligibility. When evaluating a patient for transplantation, transplant centers must consider not only the risk of cancer recurrence or de novo malignancy, but also other potential complications associated with long-term dialysis, like cardiovascular disease. Thus, the decision to waitlist a patient should be a shared decision-making process, involving transplant health professionals, the patient, and their family, taking into consideration the risk of cancer recurrence and post-transplant mortality [44], the impact of immunosuppressive therapy, as well as the patients’ values, views, and preferences as thoughtfully shown by Wong and Lim [45].

## Figures and Tables

**Table 1:** Descriptive summary table of groups of patients with and without cancer.

	[ALL]	No Cancer	Cancer	*p* Value
	N = 5879	N = 5122	N = 757	
Age (years)	65.2 (14.2)	64.5 (14.6)	69.8 (10.7)	<0.001
gender: male (%)	3519 (59.9%)	3036 (59.3%)	483 (63.8%)	0.020
Age > 65 years (%)	3292 (56.0%)	2757 (53.8%)	535 (70.7%)	<0.001
Body mass index (kg/m^2^)	27.5 (6.02)	27.6 (6.10)	27.1 (5.40)	0.029
Charlson Comorbidity Index score	4.17 (1.90)	3.87 (1.70)	6.16 (2.01)	<0.001
Metastatic solid tumor (n)	45 (0.77%)	0 (0.00%)	45 (5.94%)	<0.001
Cardiovascular disease (n)	3564 (60.6%)	3078 (60.1%)	486 (64.2%)	0.034
Diabetes (n)	2068 (35.2%)	1810 (35.3%)	258 (34.1%)	0.526
Time on dialysis (months)	59.6 (64.0)	59.8 (64.8)	57.9 (58.6)	0.425
Effective weekly treatment time (min/week)	702 (115)	702 (115)	703 (110)	0.796
eKT/V	1.37 (0.31)	1.37 (0.32)	1.38 (0.27)	0.214
Ultrafiltration during dialysis (mL/h)	2069 (914)	2085 (916)	1962 (892)	<0.001
Vascular access: arterio-venous fistula (n)	3325 (56.6%)	2895 (56.5%)	430 (56.8%)	0.915
Hypertension (n)	5330 (90.7%)	4646 (90.7%)	684 (90.4%)	0.809
Hypotensive drugs (n)	2.33 (1.51)	2.34 (1.52)	2.26 (1.44)	0.154
Angiotensin converting enzyme inhibitors (n)	1463 (24.9%)	1284 (25.1%)	179 (23.6%)	0.424
Angiotensin 2 receptor blockers (n)	538 (9.15%)	465 (9.08%)	73 (9.64%)	0.663
Calcium channel blockers (n)	2651 (45.1%)	2322 (45.3%)	329 (43.5%)	0.354
Beta blockers (n)	3825 (65.1%)	3347 (65.3%)	478 (63.1%)	0.252
Diuretics (n)	3200 (54.4%)	2795 (54.6%)	405 (53.5%)	0.609
Other hypotensive drugs (n)	1988 (33.8%)	1746 (34.1%)	242 (32.0%)	0.267
Systolic blood pressure before dialysis (mmHg)	141 (17.0)	141 (17.0)	138 (17.1)	<0.001
Diastolic blood pressure before dialysis (mmHg)	76.2 (9.71)	76.5 (9.71)	74.0 (9.45)	<0.001
Heart rate (beats/min)	74.0 (8.51)	74.0 (8.44)	74.2 (8.96)	0.536
Phosphate (mg/dL)	4.79 (1.59)	4.83 (1.60)	4.54 (1.44)	<0.001
PTH (pg/m)	384 (391)	392 (399)	328 (330)	<0.001
Albumin (g/dL)	3.89 (0.49)	3.90 (0.49)	3.81 (0.54)	<0.001
Lean tissue mass (kg)	34.9 (10.7)	35.0 (10.8)	34.2 (9.74)	0.037
Adipose tissue mass (kg)	39.5 (17.4)	39.5 (17.6)	39.3 (16.7)	0.742
Fat tissue mass (kg)	29.0 (12.8)	29.1 (12.9)	28.9 (12.2)	0.741
Lean tissue index (kg/m^2^)	12.6 (3.16)	12.6 (3.19)	12.3 (2.95)	0.005
Relative lean tissue mass (%)	46.6 (14.5)	46.6 (14.6)	46.0 (13.7)	0.276
Relative fat tissue mass (%)	36.6 (11.0)	36.6 (11.1)	36.8 (10.6)	0.678
Hemoglobin (g/dL)	10.8 (1.36)	10.8 (1.35)	10.6 (1.41)	<0.001
Ferritin (µg/dL)	691 (696)	654 (609)	918 (1061)	<0.001
MCHC (g/dL)	34.5 (78.6)	34.8 (84.2)	32.7 (1.37)	0.084
MCV (f/L)	96.1 (6.56)	96.0 (6.57)	96.8 (6.46)	0.001
Alkaline phosphatase activity (U/L)	103 (88.8)	102 (85.2)	106 (109)	0.361
Corrected calcium level (mmol/L)	9.07 (0.79)	9.07 (0.79)	9.06 (0.79)	0.830
Diabetic nephropathy (n)	646 (11.0%)	589 (11.5%)	57 (7.53%)	0.00
Glomerulonephritis (n)	498 (8.47%)	464 (9.06%)	34 (4.49%)	<0.001
Polycystic kidney disease (n)	327 (5.56%)	304 (5.94%)	23 (3.04%)	0.002
Vascular disease/hypertension (n)	473 (8.05%)	413 (8.06%)	60 (7.93%)	0.954
Miscellaneous (n)	2867 (48.8%)	2468 (48.2%)	399 (52.7%)	0.022

Data given are means ± SD. eKt/V—estimated urea kinetic clearance over time, PTH—parathyroid hormone, MCHC—mean corpuscular hemoglobin content, MCV—mean corpuscular volume.

**Table 2:** Descriptive summary of patients with malignancy in relation to age.

	[ALL]	<65 Years Old	>65 Years Old	*p* Value
	N = 757	N = 222	N = 535	
Age (years)	69.8 (10.7)	57.2 (8.38)	75.1 (6.22)	<0.001
Gender: male (%)	483 (63.8%)	137 (61.7%)	346 (64.7%)	0.491
Body mass index (kg/m^2^)	27.1 (5.40)	27.0 (5.99)	27.2 (5.14)	0.778
Charlson Comorbidity Index score	6.16 (2.01)	5.74 (2.02)	6.33 (1.98)	<0.001
Metastatic solid tumor (n)	45 (5.94%)	14 (6.31%)	31 (5.79%)	0.918
Cardiovascular disease (n)	486 (64.2%)	108 (48.6%)	378 (70.7%)	<0.001
Diabetes (n)	258 (34.1%)	60 (27.0%)	198 (37.0%)	0.011
Time on dialysis (months)	57.9 (58.6)	65.8 (72.8)	54.7 (51.4)	0.041
Effective weekly treatment time (min/week)	703 (110)	694 (142)	707 (93.5)	0.228
eKT/V	1.38 (0.27)	1.36 (0.30)	1.39 (0.26)	0.321
Ultrafiltration during dialysis (mL/h)	1962 (892)	2184 (996)	1870 (828)	<0.001
Vascular access: arterio-venous fistula (n)	430 (56.8%)	128 (57.7%)	302 (56.4%)	0.822
Hypertension (n)	684 (90.4%)	195 (87.8%)	489 (91.4%)	0.168
Hypotensive drugs (n)	2.26 (1.44)	2.32 (1.54)	2.23 (1.40)	0.487
Angiotensin converting enzyme inhibitors (n)	179 (23.6%)	51 (23.0%)	128 (23.9%)	0.852
Angiotensin 2 receptor blockers (n)	73 (9.64%)	27 (12.2%)	46 (8.60%)	0.168
Calcium channel blockers (n)	329 (43.5%)	106 (47.7%)	223 (41.7%)	0.146
Beta blockers (n)	478 (63.1%)	141 (63.5%)	337 (63.0%)	0.958
Diuretics (n)	405 (53.5%)	105 (47.3%)	300 (56.1%)	0.034
Other hypotensive drugs (n)	242 (32.0%)	83 (37.4%)	159 (29.7%)	0.048
Systolic blood pressure before dialysis (mmHg)	138 (17.1)	141 (16.4)	137 (17.3)	0.012
Diastolic blood pressure before dialysis (mmHg)	74.0 (9.45)	78.2 (9.44)	72.3 (8.90)	<0.001
Heart rate (beats/min)	74.2 (8.96)	75.7 (9.34)	73.5 (8.73)	0.003
Phosphate [mg/dl]	4.54 (1.44)	4.89 (1.62)	4.39 (1.33)	<0.001
PTH [pg/mL]	328 (330)	395 (386)	300 (299)	0.001
Albumin (g/dL)	3.81 (0.54)	3.85 (0.59)	3.80 (0.51)	0.245
Lean tissue mass (kg)	34.2 (9.74)	37.0 (10.3)	33.1 (9.28)	<0.001
Adipose tissue mass (kg)	39.3 (16.7)	37.7 (18.6)	39.9 (15.8)	0.152
Fat tissue mass (kg)	28.9 (12.2)	27.7 (13.7)	29.4 (11.6)	0.151
Lean tissue index (kg/m^2^)	12.3 (2.95)	13.0 (2.95)	12.0 (2.91)	<0.001
Fat tissue index [kg/m^2^]	14.3 (6.14)	13.3 (6.35)	14.7 (6.02)	0.011
Relative lean tissue mass (%)	46.0 (13.7)	49.5 (13.9)	44.7 (13.4)	<0.001
Relative fat tissue mass (%)	36.8 (10.6)	34.4 (11.0)	37.7 (10.2)	<0.001
Hemoglobin (g/dL)	10.6 (1.41)	10.5 (1.54)	10.7 (1.35)	0.217
Ferritin (µg/dL)	918 (1061)	982 (801)	892 (1151)	0.472
MCHC (g/dL)	32.7 (1.37)	32.9 (1.27)	32.7 (1.41)	0.029
MCV (f/L)	96.8 (6.46)	96.2 (7.22)	97.1 (6.09)	0.122
Alkaline phosphatase activity (U/L)	106 (109)	111 (109)	105 (110)	0.502
Corrected calcium level (mmol/L)	9.06 (0.79)	9.03 (0.84)	9.08 (0.77)	0.414
Diabetic nephropathy (n)	57 (7.53%)	16 (7.21%)	41 (7.66%)	0.948
Glomerulonephritis (n)	34 (4.49%)	19 (8.56%)	15 (2.80%)	0.001
Polycystic kidney disease (n)	23 (3.04%)	8 (3.60%)	15 (2.80%)	0.725
Vascular disease/hypertension (n)	60 (7.93%)	12 (5.41%)	48 (8.97%)	0.132
Miscellaneous (n)	399 (52.7%)	113 (50.9%)	286 (53.5%)	0.574

Data given are means ± SD. eKt/V—estimated urea kinetic clearance over time, PTH—parathyroid hormone, MCHC—mean corpuscular hemoglobin content, MCV—mean corpuscular volume.

**Table 3:** Descriptive summary of patients with malignancy in relation to gender.

	[ALL]	Female	Male	*p* Value
	N = 757	N = 274	N = 483	
Age (years, n)	69.8 (10.7)	69.5 (10.8)	70.0 (10.6)	0.490
Age over 65 years (n)	535 (70.7%)	189 (69.0%)	346 (71.6%)	0.491
Body mass index (kg/m^2^)	27.1 (5.40)	27.3 (6.05)	27.0 (5.00)	0.522
Charlson Comorbidity Index score	6.16 (2.01)	6.04 (2.02)	6.22 (2.00)	0.238
Metastatic solid tumor (n)	45 (5.94%)	19 (6.93%)	26 (5.38%)	0.479
Cardiovascular disease (n)	486 (64.2%)	158 (57.7%)	328 (67.9%)	0.006
Diabetes (n)	258 (34.1%)	89 (32.5%)	169 (35.0%)	0.535
Time on dialysis (months)	57.9 (58.6)	62.8 (65.3)	55.2 (54.3)	0.104
Effective weekly treatment time (min/week)	703 (110)	700 (104)	704 (114)	0.630
eKT/V	1.38 (0.27)	1.51 (0.27)	1.31 (0.23)	<0.001
Ultrafiltration during dialysis (mL/h)	1962 (892)	1816 (792)	2045 (934)	<0.001
Vascular access: arterio-venous fistula (n)	430 (56.8%)	143 (52.2%)	287 (59.4%)	0.064
Hypertension (n)	684 (90.4%)	245 (89.4%)	439 (90.9%)	0.595
Hypotensive drugs (n)	2.26 (1.44)	2.18 (1.44)	2.30 (1.44)	0.244
Angiotensin converting enzyme inhibitors (n)	179 (23.6%)	60 (21.9%)	119 (24.6%)	0.445
Angiotensin 2 receptor blockers (n)	73 (9.64%)	25 (9.12%)	48 (9.94%)	0.813
Calcium channel blockers (n)	329 (43.5%)	116 (42.3%)	213 (44.1%)	0.693
Beta blockers (n)	478 (63.1%)	175 (63.9%)	303 (62.7%)	0.816
Diuretics (n)	405 (53.5%)	146 (53.3%)	259 (53.6%)	0.989
Other hypotensive drugs (n)	242 (32.0%)	73 (26.6%)	169 (35.0%)	0.022
Systolic blood pressure before dialysis (mmHg)	138 (17.1)	137 (16.8)	139 (17.2)	0.150
Diastolic blood pressure before dialysis (mmHg)	74.0 (9.45)	73.1 (8.95)	74.5 (9.70)	0.045
Heart rate (beats/min)	74.2 (8.96)	74.4 (8.20)	74.1 (9.38)	0.616
Phosphate (mg/dL)	4.54 (1.44)	4.39 (1.31)	4.63 (1.51)	0.023
PTH (g/m)	328 (330)	353 (381)	314 (296)	0.148
Albumin (g/dL)	3.81 (0.54)	3.81 (0.53)	3.81 (0.54)	0.958
Lean tissue mass (kg)	34.2 (9.74)	28.2 (6.57)	37.7 (9.58)	<0.001
Adipose tissue mass (kg)	39.3 (16.7)	39.8 (17.4)	39.0 (16.2)	0.539
Fat tissue mass (kg)	28.9 (12.2)	29.3 (12.8)	28.7 (11.9)	0.541
Lean tissue index (kg/m^2^)	12.3 (2.95)	11.2 (2.36)	12.9 (3.08)	<0.001
Fat tissue index [kg/m^2^]	14.3 (6.14)	15.9 (6.84)	13.4 (5.49)	<0.001
Relative lean tissue mass (%)	46.0 (13.7)	42.2 (13.7)	48.3 (13.2)	<0.001
Relative fat tissue mass (%)	36.8 (10.6)	40.1 (10.6)	34.8 (10.1)	<0.001
Hemoglobin (g/dL)	10.6 (1.41)	10.4 (1.36)	10.7 (1.43)	0.004
Ferritin (µg/dL)	918 (1061)	900 (802)	930 (1199)	0.810
MCHC (g/dL)	32.7 (1.37)	32.6 (1.53)	32.8 (1.27)	0.019
MCV (f/L)	96.8 (6.46)	98.0 (6.53)	96.2 (6.33)	<0.001
Alkaline phosphatase activity (U/L)	106 (109)	114 (107)	102 (111)	0.145
Corrected calcium level (mmol/L)	9.06 (0.79)	9.13 (0.81)	9.03 (0.78)	0.112
Diabetic nephropathy (n)	57 (7.53%)	23 (8.39%)	34 (7.04%)	0.592
Glomerulonephritis (n)	34 (4.49%)	13 (4.74%)	21 (4.35%)	0.944
Polycystic kidney disease (n)	23 (3.04%)	14 (5.11%)	9 (1.86%)	0.023
Vascular disease/hypertension (n)	60 (7.93%)	17 (6.20%)	43 (8.90%)	0.238
Miscellaneous (n)	399 (52.7%)	141 (51.5%)	258 (53.4%)	0.658

Data given are means ± SD. eKt/V—estimated urea kinetic clearance over time, PTH—parathyroid hormone, MCHC—mean corpuscular hemoglobin content, MCV—mean corpuscular volume.

**Table 4:** Descriptive summary of patients with malignancy with and without metastatic solid tumors.

	[ALL]	Without	With	*p* Value
	N = 757	N = 712	N = 45	
Age (years)	69.8 (10.7)	69.8 (10.4)	69.5 (14.0)	0.889
Gender: male (n)	483 (63.8%)	457 (64.2%)	26 (57.8%)	0.479
Age over 65 years (n)	535 (70.7%)	504 (70.8%)	31 (68.9%)	0.918
Body mass index (kg/m^2^)	27.1 (5.40)	27.3 (5.39)	24.9 (5.14)	0.005
Charlson Comorbidity Index score	6.16 (2.01)	5.85 (1.59)	11.0 (1.67)	<0.001
Cardiovascular disease (n)	486 (64.2%)	459 (64.5%)	27 (60.0%)	0.656
Diabetes (n)	258 (34.1%)	239 (33.6%)	19 (42.2%)	0.305
Time on dialysis (months)	57.9 (58.6)	58.8 (59.5)	44.6 (41.4)	0.038
Effective weekly treatment time (min/week)	703 (110)	704 (109)	681 (121)	0.216
eKT/V	1.38 (0.27)	1.38 (0.26)	1.35 (0.33)	0.533
Ultrafiltration during dialysis (mL/h)	1962 (892)	1975 (892)	1763 (874)	0.122
Vascular access: arterio-venous fistula (n)	430 (56.8%)	401 (56.3%)	29 (64.4%)	0.362
Hypertension (n)	684 (90.4%)	647 (90.9%)	37 (82.2%)	0.067
Hypotensive drugs (n)	2.26 (1.44)	2.28 (1.44)	1.91 (1.53)	0.125
Angiotensin converting enzyme inhibitors (n)	179 (23.6%)	174 (24.4%)	5 (11.1%)	0.063
Angiotensin 2 receptor blockers (n)	73 (9.64%)	69 (9.69%)	4 (8.89%)	1.000
Calcium channel blockers (n)	329 (43.5%)	306 (43.0%)	23 (51.1%)	0.362
Beta blockers (n)	478 (63.1%)	457 (64.2%)	21 (46.7%)	0.028
Diuretics (n)	405 (53.5%)	386 (54.2%)	19 (42.2%)	0.159
Other hypotensive drugs (n)	242 (32.0%)	228 (32.0%)	14 (31.1%)	1.000
Systolic blood pressure before dialysis (mmHg)	138 (17.1)	139 (17.1)	134 (16.5)	0.081
Diastolic blood pressure before dialysis (mmHg)	74.0 (9.45)	74.1 (9.44)	72.4 (9.60)	0.262
Heart rate (beats/min)	74.2 (8.96)	74.1 (8.85)	75.6 (10.6)	0.339
Phosphate (mg/dL)	4.54 (1.44)	4.55 (1.45)	4.39 (1.37)	0.472
PTH (pg/mL)	328 (330)	327 (329)	343 (335)	0.764
Albumin (g/dL)	3.81 (0.54)	3.82 (0.53)	3.68 (0.61)	0.153
Lean tissue mass (kg)	34.2 (9.74)	34.4 (9.80)	30.9 (8.31)	0.015
Adipose tissue mass (kg)	39.3 (16.7)	39.7 (16.6)	33.9 (16.0)	0.031
Fat tissue mass (kg)	28.9 (12.2)	29.2 (12.2)	24.9 (11.7)	0.031
Lean tissue index (kg/m^2^)	12.3 (2.95)	12.3 (2.97)	11.5 (2.57)	0.066
Fat tissue index [kg/m^2^]	14.3 (6.14)	14.4 (6.14)	12.7 (5.92)	0.093
Relative lean tissue mass (%)	46.0 (13.7)	46.0 (13.7)	47.1 (13.6)	0.601
Relative fat tissue mass (%)	36.8 (10.6)	36.9 (10.6)	35.3 (10.5)	0.356
Hemoglobin (g/dL)	10.6 (1.41)	10.7 (1.39)	9.95 (1.59)	0.006
Ferritin (µg/dL)	918 (1061)	918 (1082)	918 (596)	0.997
MCHC (g/dL)	32.7 (1.37)	32.8 (1.37)	32.5 (1.35)	0.223
MCV (f/L)	96.8 (6.46)	96.8 (6.41)	97.4 (7.20)	0.568
Alkaline phosphatase activity (U/L)	106 (109)	103 (87.8)	163 (282)	0.178
Corrected calcium level (mmol/L)	9.06 (0.79)	9.07 (0.80)	8.91 (0.72)	0.161
Diabetic nephropathy (n)	57 (7.53%)	55 (7.72%)	2 (4.44%)	0.568
Glomerulonephritis (n)	34 (4.49%)	32 (4.49%)	2 (4.44%)	1.000
Polycystic kidney disease (n)	23 (3.04%)	22 (3.09%)	1 (2.22%)	1.000
Vascular disease/hypertension (n)	60 (7.93%)	58 (8.15%)	2 (4.44%)	0.569
Miscellaneous (n)	399 (52.7%)	370 (52.0%)	29 (64.4%)	0.141

Data given are means ± SD. eKt/V—estimated urea kinetic clearance over time, PTH—parathyroid hormone, MCHC—mean corpuscular hemoglobin content, MCV—mean corpuscular volume.

**Table 5:** Descriptive summary table by groups in relation to their status in the waiting list.

	[ALL]	Waitlisted	Not Waitlisted	*p* Value
	N = 708	N = 27	N = 681	
Age (years)	70.1 (10.5)	62.9 (8.74)	70.4 (10.4)	<0.001
Gender: male (%)	454 (64.1%)	15 (55.6%)	439 (64.5%)	0.458
Age > 65 years (%)	509 (71.9%)	12 (44.4%)	497 (73.0%)	0.003
Body mass index (kg/m^2^)	27.2 (5.42)	26.3 (5.17)	27.2 (5.43)	0.387
Charlson Comorbidity Index score	6.18 (2.00)	5.48 (1.78)	6.21 (2.00)	0.047
Cardiovascular disease (n)	462 (65.3%)	19 (70.4%)	443 (65.1%)	0.716
Diabetes (n)	241 (34.0%)	3 (11.1%)	238 (34.9%)	0.018
Time on dialysis (months)	59.5 (58.5)	70.8 (50.6)	59.0 (58.8)	0.248
Effective weekly treatment time (min/week)	709 (94.4)	723 (59.8)	709 (95.5)	0.246
eKT/V	1.39 (0.26)	1.40 (0.30)	1.39 (0.26)	0.851
Ultrafiltration during dialysis (mL/h)	1972 (891)	2105 (804)	1967 (895)	0.392
Vascular access: arterio-venous fistula (n)	413 (58.3%)	17 (63.0%)	396 (58.1%)	0.765
Hypertension (n)	647 (91.4%)	27 (100%)	620 (91.0%)	0.158
Hypotensive drugs (n)	2.30 (1.42)	2.52 (1.83)	2.29 (1.40)	0.530
Angiotensin converting enzyme inhibitors (n)	174 (24.6%)	9 (33.3%)	165 (24.2%)	0.395
Angiotensin 2 receptor blockers (n)	69 (9.75%)	3 (11.1%)	66 (9.69%)	0.740
Calcium channel blockers (n)	311 (43.9%)	16 (59.3%)	295 (43.3%)	0.150
Beta blockers (n)	456 (64.4%)	16 (59.3%)	440 (64.6%)	0.715
Diuretics (n)	389 (54.9%)	11 (40.7%)	378 (55.5%)	0.188
Other hypotensive drugs (n)	228 (32.2%)	13 (48.1%)	215 (31.6%)	0.110
Systolic blood pressure before dialysis (mmHg)	139 (16.7)	146 (12.0)	138 (16.8)	0.005
Diastolic blood pressure before dialysis (mmHg)	73.9 (9.41)	77.7 (6.96)	73.8 (9.47)	0.009
Heart rate (beats/min)	74.0 (8.79)	72.3 (5.37)	74.1 (8.89)	0.115
Phosphate (mg/dL)	4.52 (1.43)	5.20 (1.19)	4.49 (1.43)	0.005
PTH (pg/mL)	327 (327)	642 (640)	315 (303)	0.016
Albumin (g/dL)	3.83 (0.50)	3.96 (0.35)	3.83 (0.50)	0.079
Lean tissue mass (kg)	34.1 (9.67)	34.6 (8.97)	34.1 (9.70)	0.797
Adipose tissue mass (kg)	39.4 (16.7)	37.4 (15.6)	39.5 (16.8)	0.512
Fat tissue mass (kg)	29.0 (12.3)	27.5 (11.5)	29.0 (12.3)	0.515
Lean tissue index (kg/m^2^)	12.3 (2.93)	12.4 (2.20)	12.3 (2.96)	0.734
Relative lean tissue mass (%)	46.0 (13.8)	47.5 (12.6)	45.9 (13.9)	0.552
Relative fat tissue mass (%)	36.8 (10.6)	35.8 (10.8)	36.9 (10.6)	0.624
Hemoglobin (g/dL)	10.7 (1.40)	10.5 (1.29)	10.7 (1.41)	0.523
Ferritin (µg/dL)	935 (1074)	620 (361)	946 (1090)	0.034
MCHC (g/dL)	32.7 (1.38)	32.6 (1.01)	32.8 (1.39)	0.543
MCV (f/L)	97.0 (6.30)	95.5 (3.68)	97.0 (6.38)	0.046
Alkaline phosphatase activity (U/L)	105 (107)	159 (231)	103 (98.9)	0.227
Corrected calcium level (mmol/L)	9.06 (0.79)	8.89 (0.93)	9.07 (0.78)	0.337
Diabetic nephropathy (n)	53 (7.49%)	1 (3.70%)	52 (7.64%)	0.713
Glomerulonephritis (n)	33 (4.66%)	4 (14.8%)	29 (4.26%)	0.032
Polycystic kidney disease (n)	21 (2.97%)	2 (7.41%)	19 (2.79%)	0.189
Vascular disease/hypertension (n)	60 (8.47%)	4 (14.8%)	56 (8.22%)	0.276
Miscellaneous (n)	380 (53.7%)	10 (37.0%)	370 (54.3%)	0.116

Data given are means ± SD. eKt/V—estimated urea kinetic clearance over time, PTH—parathyroid hormone, MCHC—mean corpuscular hemoglobin content, MCV—mean corpuscular volume.

## Data Availability

The data underlying this article will be shared upon reasonable request to the corresponding author.
